# Influence of Mediterranean Diet Adherence and Physical Activity on Bone Health in Celiac Children on a Gluten-Free Diet

**DOI:** 10.3390/nu13051636

**Published:** 2021-05-13

**Authors:** Teresa Nestares, Rafael Martín-Masot, Carlos de Teresa, Rocío Bonillo, José Maldonado, Marta Flor-Alemany, Virginia A. Aparicio

**Affiliations:** 1 Department of Physiology, Faculty of Pharmacy, University of Granada, 18071 Granada, Spain; floralemany@ugr.es (M.F.-A.); virginiaparicio@ugr.es (V.A.A.); 2 Biomedical Research Centre (CIBM), Institute of Nutrition and Food Technology “José MataixVerdú” (INYTA), University of Granada, 18071 Granada, Spain; 3 Pediatric Gastroenterology and Nutrition Unit, Hospital Regional Universitario de Malaga, 29010 Málaga, Spain; rafammgr@gmail.com; 4 Andalusia Sport Medicine Centre, 18007 Granada, Spain; cdeteresa2403@gmail.com; 5 Department of Pediatrics, University of Granada, 18071 Granada, Spain; jmaldon@ugr.es; 6 Pediatric Gastroenterology and Nutrition Unit, Hospital Universitario Virgen de las Nieves, 18071 Granada, Spain; 7 Spain Maternal and Child Health Network, Carlos III Health Institute, 28029 Madrid, Spain; 8 Sport and Health University Research Institute (IMUDS), 18007 Granada, Spain

**Keywords:** celiac disease, gluten-free diet, Mediterranean Diet, children, body composition

## Abstract

We aimed to assess the influence of the Mediterranean Diet adherence and physical activity (PA) on body composition, with a particular focus on bone health, in young patients with celiac disease (CD). The CD group (*n* = 59) included children with CD with a long (>18 months, *n* = 41) or recent (<18 months, *n* = 18) adherence to a gluten-free diet (GFD). The non-celiac group (*n* = 40) included non-celiac children. After adjusting for potential confounders, the CD group showed lower body weight (*p* = 0.034), lean mass (*p* = 0.003), bone mineral content (*p* = 0.006), and bone Z-score (*p* = 0.036) than non-celiac children, even when the model was further adjusted for adherence to a GFD for at least 18 months. Among CD children, spending greater time in vigorous physical activity was associated with higher lean mass (*p* = 0.020) and bone mineral density with evidence of statistical significance (*p* = 0.078) regardless of the time they followed a GFD. In addition, a greater Mediterranean Diet adherence was associated with a higher bone Z-score (*p* = 0.020). Moreover, lean mass was strongly associated with bone mineral density and independently explained 12% of its variability (*p* < 0.001). These findings suggest the importance of correctly monitoring lifestyle in children with CD regarding dietary habits and PA levels to improve lean mass and, consequently, bone quality in this population.

## 1. Introduction

Celiac disease (CD) is a multifactorial, systemic autoimmune disorder. The ingestion of gluten and related proteins triggers an immune reaction causing CD in genetically susceptible individuals. This disease occurs in around 1–1.4% of individuals from most populations [1]. Although the prevalence of CD has increased in developed countries over recent decades, the ratio between diagnosed and undiagnosed cases ranges between 1:3 and 1:5; that is, a high number of cases are undetected [2]. This fact can be critical in a stage of maximum growth and use of nutrients such as childhood, since problems such as growth failure and incorrect bone formation can occur, even in situations of silent CD.

In recent years, the alteration of bone mass or low bone mineral density (BMD) has been the subject of attention since the bone mass acquired at the end of development is a good parameter to predict the future risk of fractures [3]. Traditionally, CD has been associated with low body weight, height, and BMD. Up to 70% of patients with CD present bone alterations in adulthood [4] and, consequently, increased risk of fractures [5,6]. The origin of metabolic bone disease is multifactorial in CD. In addition to the poor absorption of vitamin D and calcium and the sequestration of calcium and magnesium by unabsorbed fats due to malabsorption, it seems that the persistent activation of inflammation is the leading cause of the pathophysiology of metabolic bone disease [7]. Deficiencies in some nutrients, such as minerals (calcium, zinc, iron) and vitamins (vitamin D and other fat-soluble vitamins), due to malabsorption processes inherent to this condition are common in untreated CD patients or before diagnosis [8]. 

The unique therapy for CD is consuming a strict gluten-free diet (GFD) for someone’s entire life: it decreases complications and is beneficial for health [9]. Indeed, a GFD has been shown to improve BMD in patients with CD [10,11,12,13], suggesting that early diagnosis of the disease could protect celiac patients from osteoporosis [14]. Once patients adhere to the GFD, the intestinal atrophy is restored, so nutrients are adequately absorbed [15]. The assessment of the effect of a GFD on children’s growth is required to ensure adequate growth and pubertal development [16]. Nevertheless, inconsistent results about the nutritional adequacy of the GFD have been reported. Various studies suggest that GFD is nutritionally unbalanced because its micronutrient content (e.g., calcium, iron, vitamin D) is lower than recommended [8,17,18,19,20]. Therefore, additional factors must be taken into consideration. Firstly, the nutritional adequacy of GFD is critical for suitable treatment and prevention of additional problems derived from the oxidative stress and inflammation promoted by CD [20], which might also be involved in the pathophysiology of metabolic bone disease [7]. It is also essential to ensure that the GFD follows the Mediterranean Diet (MD) because this healthy dietary pattern involves a high content of nutrients that could be important to achieve a healthy musculoskeletal system [21], and it is associated with a rise in BMD and prevention of osteoporosis [22,23].

Children recently diagnosed with CD should adhere strictly to the GFD to normalize their weight and BMD, but the reality is that this is not always achieved. It is recommended that bone health be periodically assessed in children with CD, at least until growth detention [24]. Previous studies suggest that potentially modifiable factors might positively impact BMD; however, these results have not been fully verified. In this context, adequate physical activity (PA) levels during childhood are essential for bone formation and for achieving an optimal bone peak mass [25]. Usually, PA levels are not assessed despite their importance for BMD and general health status. Higher levels of PA or exercise, especially at moderate–vigorous intensities and including resistance and impact activities, may promote higher BMD [26,27]. Osteoporosis has become a primary global public health concern. In children and adolescents, weight-bearing PA contributes to reaching the maximum peak of bone mass at these life stages, resulting in stronger and healthier bones that prevent osteoporosis because of the consequent lower risk of fragility fractures in adulthood and the elderly [28]. Childhood is a considerably critical stage in the bone health of adults. Therefore, further characterization of the extent to which these modifiable factors (diet quality and PA levels) might be associated with BMD in children with CD has clinical and public health interest. This characterization may be relevant for implementing successful programs to prevent bone loss in this specific population. Therefore, the aims of the present study were: (1) to assess the association of the MD adherence and PA with various components of body composition, with a particular focus on bone health, in children with CD and, (2) to evaluate which of these modifiable factors (MD adherence or PA) are independently associated with BMD in children with CD.

## 2. Materials and Methods 

### 2.1. Subjects

This prospective cross-sectional study was approved by the Ethics Committee of the University of Granada (Ref. 201202400000697). It was performed according to the Declaration of Helsinki principles and its later amendments. 

A total of 40 non-celiac children comprised the non-celiac group. They do not present a history of chronic disease and showed a negative serological screening. The non-celiac children attended this service because of minor symptoms related to chronic functional constipation, based on the Rome IV criteria [29]. They were included in the non-celiac disease group after confirming that they only had transitory gastrointestinal symptoms (functional constipation). The inclusion criteria for the non-celiac group were: 7–18 years old, normal weight for the age and appetite, absence of serum IgA and IgG anti-transglutaminase (tTG) antibodies, and no gastrointestinal disorders in the previous year.

The study also included 59 children aged from 7 to 18 years old (CD group); these children attended the Gastroenterology, Hepatology and Child Nutrition Service from the “Virgen de las Nieves” University Hospital in Granada (Spain). The CD group (*n* = 59) consisted of children with CD diagnosed according to the European Society for Pediatric Gastroenterology Hepatology and Nutrition (ESPGHAN) [30]. 

The exclusion criteria for both groups were kidney or liver diseases, diabetes, acute and chronic inflammation, inflammatory bowel disease, chronic asthma, and consumption of dietary supplements containing substances with antioxidant activity. Obese patients (according to the International Task Force criteria) [31] and those refusing to sign the informed consent were excluded too. Written informed consent was obtained from all parents.

### 2.2. Clinical and Sociodemographic Characteristics

An initial survey (anamnesis) was performed to compile information on the sociodemographic and clinical features of the study participants (e.g., age, sex, time following a gluten-free diet, diagnosis of other diseases, and marital status of their parents).

### 2.3. Physical Activity Assessment

Physical activity was registered using the International Physical Activity Questionnaire (IPAQ) [32]. It includes some questions on the PA performed in the last week. A score from 0 to 5 was assigned using this questionnaire. The intensity and frequency of each activity were considered, so quantitative and qualitative assessment of PA was performed. Moderate and vigorous physical activity levels (min/day) were calculated. A dichotomous variable to indicate if children met the World Health Organization PA guidelines [33] was created, using a cut-off of a minimum of 60 min per day of moderate to vigorous physical activity and incorporation of vigorous physical activity at least three days per week. 

### 2.4. Anthropometric Measures

A scale (InBody R20, Biospace, Seoul, Korea) and a stadiometer (Seca 22, Hamburg, Germany) were used to assess weight (kg) and height (cm), respectively. Both variables were used to calculate the body mass index (BMI) (weight [kg]/height [m^2^]). 

### 2.5. Bone Mineral Density and Body Composition

Lean fat and bone mass of the whole body were measured using a dual-energy X-ray absorptiometry (DXA) device (DXA; Discovery Wi, Hologic, Bedford, MA, USA) that meets the conditions required for study in childhood. Measurements were performed in the posteroanterior spine, whereas the hip and proximal area of the femur were ignored given the great variability of these areas during growth, following the Recommendations of the International Society of Clinical Densitometry [34]. The results were interpreted by experts and expressed as a Z-score, which defines the number of standard deviations of a child’s BMD compared to the average BMD of other children of his age, height, sex, and ethnicity.

### 2.6. Dietary Assessment

The adherence to the MD was evaluated using the Mediterranean Diet Quality Index in Children and Adolescents (KIDMED) survey [35]. This index is calculated using a 16-question test that values different dietary habits. A value of +1 or −1 is assigned to each question depending on whether it meets or not the Mediterranean dietary recommendations, respectively. The KIDMED index ranges from 0 (no adherence to the MD) to 12 (complete adherence to the MD). Scores are added up to quantify the total index of the participant’s adherence to the MD. Participants are classified into 3 categories: (1) having an optimal MD adherence (≥8 points), (2) improvement needed to adjust intake to Mediterranean patterns (4–7 points), and (3) very low diet quality (≤3 points) [35].

### 2.7. Statistical Analyses

Descriptive statistics (mean (standard deviation) for quantitative variables and the number of children (%) for categorical variables) were used to describe participants’ sociodemographic and clinical characteristics (Table 1). 

A one-way analysis of covariance (ANCOVA) was used to assess the differences in body composition, bone-related variables, MD adherence, and PA levels between the CD and non-celiac groups (Table 2). Two separate models were included: Model one was adjusted for age and sex. Model two was also adjusted for adhering to a GFD for at least 18 months. 

Differences in body composition parameters among all children based on PA levels (not meeting versus meeting PA recommendations) were assessed with an ANCOVA after adjusting for age, sex, height, and following a GFD for at least 18 months (Supplementary Table S1). 

Linear regression analyses were performed to explore the association between MD adherence, moderate PA, vigorous PA, and body composition in CD group (Table 3 and Table 4). We included two separate models considering the abovementioned covariables. 

A forward stepwise regression analysis was performed including BMD as a dependent variable (Table 5) to explore potential factors affecting bone health in CD children, as this population is at higher risk of lower bone mass deposition. Age and sex were included as cofounders and kept fixed into the model (step 1). In step 2, BMI (kg/m^2^), MD adherence, lean mass (kg), moderate PA (min/day), and vigorous PA (min/day) were included in the model at the same time by using a stepwise procedure since these factors have been previously related to better bone health. Thus, the investigated components are included into the model step by step (when *p* < 0.05) based on the strength of their association with the outcome.

All analyses were performed using the Statistical Package for Social Sciences (IBM SPSS Statistics for Windows, version 22.0, Armonk, NY, USA); the significance level was set at *p* < 0.05.

## 3. Results

The baseline sociodemographic and clinical features of the study subjects are shown in Table 1. A total of 59 children with CD participated in the study (mean age 10.0 ± 3.3 years). More than 50% of the participants with CD adhered to a GFD for at least 18 months and did not have any other diseases. The non-celiac group included 40 non-celiac children (mean age 11.2 ± 3.9 years). A higher prevalence of CD among girls was found (*p* = 0.012), which is consistent with the currently available literature that indicates that girls have two to three times higher odds of having CD [1]. No other differences between groups were found in sociodemographic characteristics or PA levels (all, *p* > 0.05). 

Differences in body composition and MD adherence between the CD group and the non-celiac group are shown in Table 2. After adjusting for age, sex, and following a GFD for at least 18 months, the CD group showed lower body weight (*p* = 0.034), lean mass (*p* = 0.003), bone mineral content (*p* = 0.006), and bone Z-score (*p* = 0.036) than the non-celiac group. 

Differences in body composition among all children based on PA levels (i.e., not meeting versus meeting PA recommendations) are shown in Supplementary Table S1. After adjusting for age, sex, and following a GFD for at least 18 months, children meeting PA recommendations showed lower body weight *(p* = 0.018), lower BMI (*p* = 0.032), lower fat mass (*p* = 0.037), and lower body fat percentage (*p* = 0.047) than children not meeting PA recommendations. 

Linear regression analysis assessing the association between PA levels (moderate and vigorous) and body composition measurements in the CD group is shown in Table 3. Spending more time performing vigorous PA was associated with higher lean mass (*p* = 0.016) and higher BMD with evidence of statistical significance (*p* = 0.079). Results remained the same after additionally adjusting for following a GFD for at least 18 months (*p* = 0.021).

Linear regression analysis assessing the association between the MD adherence and body composition in CD group is shown in Table 4. A higher MD adherence was associated with a higher bone Z-score (*p* = 0.020). This association remained significant after additional adjustment for following a GFD for at least 18 months (*p* = 0.021). MD adherence was not associated with any other body composition parameter (all, *p* > 0.05). We have further adjusted the model for calcium and vitamin D, but the results remained the same. 

The independent associations (stepwise analysis) of the MD adherence, different body composition components (i.e., lean mass and BMI), and moderate and vigorous PA levels with BMD in the CD group are shown in Table 5. Age and sex were associated with BMD (β = 0.846, *p* ≤ 0.001 and β = 0.163, *p* = 0.029; respectively) and explained 71% of its variability (step 1). In the next model (step 2), lean mass (kg) was associated with BMD (β = 0.826, *p* < 0.001) and additionally explained 12% of its variability.

## 4. Discussion

Our results indicate that the CD group present with lower body weight, lower lean mass, bone mineral content, and Z-score than the non-celiac group, regardless of sex, age, and length of time on a GFD. Moreover, a higher MD adherence and more time performing vigorous PA were associated with higher Z-score and lean mass among CD children. This highlights the need for comprehensive lifestyle interventions that include a healthy dietary pattern and adequate PA or exercise for better bone health in these patients. When further analyzing which factors (i.e., MD adherence, PA levels, and body composition) were associated with bone health, lean body mass independently explained 12% of the variability in bone mineral density. Considering that higher levels of PA or exercise, especially at vigorous intensities and including resistance and impact activities, may promote higher lean mass and BMD, future studies are warranted to explore the effectiveness of specific dietary and exercise interventions focused on improving bone health in these patients.

Traditionally, the diagnosis of CD has been associated with children and adolescents presenting low weight and height and/or growth delay and the presence of lower BMD [36]. Several studies have shown lower bone mass in children with CD than in non-celiac children, especially at diagnosis, but only a few studies have monitored bone health after diagnosis. The etiology of metabolic bone disease in CD is multifactorial. Initially, the malabsorptive status could be a fundamental cause of the deficit of calcium and vitamin D through a defect in the utilization of nutrients and the sequestration of calcium and magnesium by the non-absorbed fat. It has also been postulated that deficiency of minerals in the GFD, such as calcium and magnesium, could affect BMD [19]. Several authors [19,37,38] have described a lower vitamin D intake in subjects with CD. This situation could be reversed by implementing interventions with vitamin-D-fortified food products for patients with CD. Nevertheless, no information on vitamins and minerals is usually available for industrially manufactured gluten-free products. Hence, the dietary records of patients with CD could lead to an erroneous nutritional assessment concerning vitamins and minerals. Recent studies reported inconsistent results regarding serum vitamin D levels in patients with CD on a GFD [8]. In our study, although at the lower limit of normality, no differences in vitamin D values were found between the CD group and the non-celiac group, nor in the rest of the biochemical variables. Furthermore, our data confirmed that bone mass was independent of the length of time of evolution of the disease, which means that there are probably more factors involved. In this context, Blazina et al. [38] have pointed out that the improvement in BMD is due to an exhaustive adherence to the GFD and an overall healthier lifestyle.

Most studies show that markers of bone remodeling in children with CD could improve after following a GFD for 1–2 years (despite these markers still being lower than that of non-celiac children) [39,40,41]; however, GFD is a restrictive diet and its nutritional adequacy is still controversial because some studies have shown results indicating that a GFD is nutritionally unbalanced [17,42,43]. The literature data on the influence of GFD on anthropometric measurements of patients with CD are inconsistent. The association between a good adherence to the GFD and a favorable effect on anthropometric parameters, including a gain in lean mass, changes in BMI by reaching normal weight in children, and a rapid increase in linear growth, has been reported [44,45,46]; but some studies suggest a harmful effect of GFD on body composition and anthropometric measurements in patients with CD, with more prevalence of overweight and obesity [47,48], and in some cases ascribe these differences to the length of time on the GFD and the anthropometric measurements themselves. However, our findings suggest that diet quality matters, independently of being on a GFD for a long term, and that other lifestyle habits that may affect body composition might also be relevant in this population, as is the case of physical activity. Lionetti et al. [8] found no differences in BMI between children with CD on a GFD for more than 2 years and non-celiac children because both groups showed the same adherence rate to the MD, and the same weekly hours of physical activity, which reinforces our hypothesis.

In this sense, the MD is characterized by its high antioxidant effect and nutrigenomic modulation capacity, being a protective factor against several diseases [49]. The effect of MD on bone health and fracture risk has also been reported [50]. Its positive effect on health status has been attributed to the fact that it is a balanced diet, with high consumption of vegetables, legumes, fruits, and cereals; moderate to high fish intake; low intake of saturated lipids and high intake of unsaturated lipids, in particular, olive oil; low intake of meat, and low consumption of ultra-processed foods. However, the scientific literature data regarding bone are still inconsistent [51]. In our study, the MD adherence was associated with a better Z-score, but this association could have been mediated by a larger lean mass, as suggested by the stepwise regression analyses performed, where only lean mass was independently associated with BMD.

The persistent chronic inflammation seems to be one of the main causes of the pathophysiology of metabolic bone health in CD [52]. CD increases inflammatory markers, and inflammation plays a relevant role on bone remodeling, with C-reactive protein showing an inverse and independent dose–response relationship with BMD [53], whereas other authors found no association [54]. Therefore, the acceleration of BMD loss may be related to the malabsorption of nutrients necessary for bone growth and the increased concentration of inflammatory signaling molecules [55]. The potentially harmful effects of cytokines secreted by the inflamed intestine might considerably affect bone. In this sense, high consumption of ultra-processed foods has been directly related to the development of digestive diseases and other chronic non-communicable diseases [56] through a pro-inflammatory response and an increased intestinal permeability [57]. Our group previously reported a pro-inflammatory cytokine pattern in a population with CD with high UPF consumption and low physical activity levels [20]. Consequently, it is also necessary to implement a correct adherence to the GFD during the disease, avoiding the excessive consumption of UPF. In this context, a high adherence to the Mediterranean dietary pattern could be a helpful tool.

However, as mentioned above, our results seem to indicate that only lean mass is independently associated with BMD in these patients, explaining 11% of its variability. This finding is consistent with other studies [58] and supports the role of muscle mass on bone health [59,60]. Thus, one of the potential mechanisms behind this worse bone health in these patients could be the lower lean mass found in the CD group. It has been widely found that bone mass is higher in physically active children than in less active ones [27], which is consistent with our findings, as CD children who spent more time in vigorous PA showed greater lean mass. Thus, we hypothesize that higher vigorous PA levels and better adherence to the MD could promote a higher lean mass and, consequently, better bone health. Several studies examining the role of fat and lean mass in BMD suggest that higher lean mass could be especially favorable for bone health [61]. The mechanisms behind the role of muscle mass on bone health are: mechanical loading [62,63], RANKL/RANK/OPG pathway regulation [64], stimulation of growth factors, and angiogenesis or the release of myokines [65] such as irisin [59], among others.

Furthermore, some studies show that exercise-induced increases in bone mass in children are maintained into adulthood, suggesting that PA habits during childhood may have long-lasting benefits on bone health [66]. The lack of quantitative dose–response studies prevents an in-depth description of an exercise program to optimize the peak bone mass in children and adolescents. Nevertheless, several small randomized controlled trials suggest that impact activities, moderate-intensity resistance training, and participation in sports involving running and jumping are all osteogenic stimuli in children. Vigorous PA, including both resistance and impact exercises (e.g., through jumping [25] or weight-bearing activities [67]), during childhood and adolescence, improves bone mineral content, BMD, and structural properties without side effects [28,67]. Therefore, this type of intervention should be implemented when possible to increase bone mass in the early stages of life, which may directly prevent bone diseases such as osteoporosis later in life, especially among children with CD [67].

## 5. Strengths and Limitations

The first limitation is that the sample size of the present study was relatively small; therefore, no causal relationships can be stablished because it is a cross-sectional study, and, consequently, our results must be interpreted with caution. Moreover, sex distribution differed by groups, with a higher prevalence of CD in girls than in boys. However, this constitutes the normal distribution of the disease by sexes [1], which is more predominant among women. In addition, the IPAQ was used to self-report the levels of physical activity rather than using accelerometry for an objective determination of these levels. Consequently, future studies are warranted to explore the differences and associations found in the present study using objectively measured physical activity levels. Furthermore, sun exposure questionnaires were not administered, and this variable could be correlated with BMD [68]. Moreover, limitations in the use of bone densitometry in childhood are related to the two-dimensional measurement of a three-dimensional structure in a growing skeleton. Finally, the non-celiac disease group was slightly smaller due to the problems found to recruit enough non-celiac participants. Therefore, it is advisable to recruit a larger number of participants to confirm the present findings.

## 6. Conclusions

Overall, our results indicate that children with CD have lower body weight, lean mass, and bone mass than non-celiac children, regardless of the length of time on a GFD. These results confirm that close monitoring of the disease is necessary, paying attention to other factors such as the adherence to a MD or vigorous PA to enhance lean mass, as they might be key factors to achieve better bone quality in this population. Efforts should be directed not only to establish a GFD and improve bone parameters at diagnosis but also to monitor diet and bone health during follow-up, with DXA being a factor to consider that can determine patients who are likely to benefit from a dietary supplement and a better long-term prognosis. Although some authors suggest that bone health assessment should be part of the routine management of children with CD [11], the use of DXA technology and determination of the most appropriate periodicity for the bone evaluation would be helpful.

## Figures and Tables

**Table 1:** Physical activity, Mediterranean Diet adherence, sociodemographic and clinical characteristics of the study participants.

Variable	Celiac Disease Group (*n* = 59)	Non-Celiac Group (*n* = 40)	*p*
Age (years)	10.0 (3.3)	11.2 (3.9)	0.096
Sex (female, *n* [%])	40 (67.8)	17 (42.5)	0.012
Physical activity (min/day) (*n* = 91)			
Sedentary time	475.4 (137.8)	473.1 (97.6)	0.934
Moderate physical activity	67.5 (43.1)	61.7 (46.9)	0.549
Vigorous physical activity	55.4 (42.2)	52.3 (43.7)	0.745
Mediterranean Diet adherence, (*n* [%])			
Low Mediterranean Diet adherence	5 (8.5)	1 (2.5)	0.416
Medium Mediterranean Diet adherence	29 (49.2)	23 (57.5)	
High Mediterranean Diet adherence	25 (42.4)	16 (40.0)	
Following a GFD for at least 18 months, (*n* [%])			
Yes	18 (30.5)	-	
No	41 (69.5)	-	
Parents’ marital status (married, *n* [%]) (*n* = 77)	43 (97.7)	33 (100.0)	0.383
Other diseases (yes, *n* [%]) (*n* = 63)	4 (11.4)	1 (3.6)	0.252

Values are shown as mean (standard deviation) unless otherwise indicated. GFD, gluten-free diet.

**Table 2:** Differences in Mediterranean Diet adherence, body composition, and some serum bone-related markers between groups (celiac disease versus non-celiac group).

	Celiac Disease Group (*n* = 59)	Non-Celiac Group (*n* = 40)	*p* ^a^	*p* ^b^
Mediterranean Diet adherence (0–12)	6.5 (0.3)	7.1 (0.3)	0.186	0.268
Body composition				
Weight (kg)	36.2 (0.8)	39.3 (1.0)	0.022	0.034
Height (cm)	140.9 (1.0)	141.9 (1.2)	0.560	0.424
Body mass index (kg/m^2^)	17.7 (0.3)	18.7 (0.3)	0.037	0.121
Fat mass (g)	10,155.5 (499.9)	10,571.6 (612.2)	0.607	0.999
Fat mass (%)	28.6 (0.8)	28.1 (0.9)	0.713	0.275
Lean mass (g)	24,058.5 (534.9)	26,538.1 (655.1)	0.005	0.003
Bone mineral content (g)	1124.3 (27.9)	1242.3 (34.2)	0.010	0.006
Bone mineral density (g/cm^2^)	0.79 (0.008)	0.80 (0.010)	0.226	0.302
Z-score *	−0.922 (0.1)	−0.423 (0.1)	0.008	0.036

Values are shown as mean (standard error). ^a^ Model adjusted for age and sex. ^b^ Model additionally adjusted for following a gluten-free diet for at least 18 months. * In model *p* ^a^ Z-score was unadjusted.

**Table 3:** Linear regression assessing the association between moderate and vigorous physical levels and body composition in children with celiac disease (*n* = 55).

	Unstandardized Coefficients	Standardized Coefficients	95% Confidence Interval (B)			
Moderate Physical Activity (min/day)	B	β	Lower Upper		*p* ^a^	*p* ^b^
Weight (kg)	0.007	0.029	−0.027	0.042	0.669	0.720
Fat mass (g)	5.196	0.052	−15.911	26.304	0.623	0.814
Lean mass (g)	10.126	0.056	−12.239	32.491	0.368	0.295
Fat mass (%)	−0.003	−0.018	−0.040	0.034	0.889	0.554
Bone mineral content (g)	0.374	0.044	−0.772	1.520	0.516	0.456
(g/cm^2^)	0.000	0.034	0.000	0.001	0.653	0.643
Z-score *	0.003	0.135	−0.003	0.009	0.322	0.366
Vigorous physical activity (min/day)						
Weight (kg)	0.010	0.037	−0.024	0.044	0.565	0.519
Fat mass (g)	−3.922	−0.038	−24.789	16.946	0.708	0.863
Lean mass (g)	25.773	0.140	4.846	46.701	0.017	0.020
Fat mass (%)	−0.019	−0.129	−0.055	0.017	0.289	0.451
Bone mineral content (g)	0.323	0.038	−0.811	1.456	0.570	0.613
Bone mineral density (g/cm^2^)	0.000	0.128	0.000	0.001	0.079	0.078
Z-score *	0.001	0.051	−0.005	0.008	0.710	0.632

^a^ Model adjusted for age and sex. ^b^ Model additionally adjusted for following a gluten-free diet for at least 18 months. * In model *p* ^a^ Z-score was unadjusted.

**Table 4:** Linear regression assessing the association between the Mediterranean Diet adherence and body composition in children with celiac disease (*n* = 59).

	Unstandardized Coefficients	Standardized Coefficients	95% Confidence Interval (B)			
	B	β	Lower	Upper	*p* ^a^	*p* ^b^
Weight (kg)	0.127	0.024	−0.540	0.794	0.704	0.717
Fat mass (g)	−7.899	−0.004	−420.1	404.3	0.970	0.919
Lean mass (g)	215.1	0.059	−208.3	638.6	0.313	0.297
Fat mass (%)	−0.244	−0.083	−0.965	0.477	0.501	0.426
Bone mineral content (g)	7.186	0.042	−14.852	29.225	0.516	0.512
Bone mineral density (g/cm^2^)	0.004	0.063	−0.005	0.012	0.389	0.400
Z-score *	0.143	0.298	0.022	0.264	0.022	0.021

^a^ Model adjusted for age and sex. ^b^ Model additionally adjusted for following a gluten-free diet for at least 18 months. * In model *p* ^a^ Z-score was unadjusted.

**Table 5:** Stepwise regression analysis assessing the independent association of Mediterranean Diet adherence and body composition components with bone mineral density in children with celiac disease (*n* = 55).

Bone Mineral Density (g/cm^2^)							
	B	β	SE	*p*	Adjusted R^2^	R^2^ Change	*p*
Step 1					0.714	0.724	<0.001
*Age*	0.032	0.846	0.003	<0.001			
*Sex*	0.042	0.163	0.019	0.029			
Step 2					0.839	0.123	<0.001
*Age*	0.004	0.115	0.005	0.368			
*Sex*	−0.012	−0.016	0.016	0.475			
*Lean mass (kg)*	0.013	0.826	0.002	<0.001			

SE, Standard Error; β, standardized regression coefficient; B, nonstandardized regression coefficient; R^2^, adjusted coefficient of determination, expressing the variability percent of the dependent variable explained by each model; R^2^ change, additional percent variability explained by the model due to the inclusion of the new term. In model 1, the excluded variables were body mass index (kg/m^2^), Mediterranean Diet adherence, lean mass (kg), moderate physical activity (min/day) and vigorous physical activity (min/day). In model 2, the excluded variables were body mass index (kg/m^2^), Mediterranean Diet adherence, moderate physical activity (min/day) and vigorous physical activity (min/day).

## Data Availability

The data presented in this study are available on request from the corresponding author.

## Supplementary Materials

The following are available online at https://www.mdpi.com/article/10.3390/nu13051636/s1, Table S1. Body composition differences among the study participants by meeting physical activity guidelines (not meeting versus meeting minimum physical activity recommendations).
