# Supplementary material for: Influence of Mediterranean Diet Adherence and Physical Activity on Bone Health in Celiac Children on a Gluten-Free Diet
Source: Nutrients. 2021 May 13;13(5):1636. doi: 10.3390/nu13051636 (PMC8152289; doi:10.3390/nu13051636)
Supplement: Supplementary file 1 [file nutrients-13-01636-s001.zip › nutrients-1188860-supplementary.pdf]

Supplementary Table S1. Body composition differences among the study participants by meeting physical activity guidelines (not meeting versus meeting minimum physical activity recommendations).

	Not meeting PA recommendations (n=41)	Meeting PA recommendations (n=50)	P[a]	P[b]
Body composition				
Weight (kg)	39.3 (0.8)	26.6 (0.7)	**0.013**	**0.018**
Body mass index (kg/m²)	18.6 (0.3)	17.6 (0.3)	**0.022**	**0.032**
Fat mass (g)	11392.8 (552.7)	9705.3 (499.8)	**0.027**	**0.037**
Fat mass (%)	29.9 (0.9)	27.4 (0.8)	**0.033**	**0.047**
Lean mass (g)	26921.7 (487.7)	24791.9 (441.0)	0.091	0.104
Bone mineral content (g)	1219.1 (29.6)	1164.9 (26.8)	0.182	0.192
Bone mineral density (g/cm²)	0.8 (0.1)	0.8 (0.1)	0.784	0.844
*Z-score**	-0.6 (0.1)	-0.7 (0.1)	0.594	0.519

Values are shown as mean (standard error). PA, physical activity. [a]Model adjusted for age, sex and height. [b]Model additionally adjusted for following a gluten-free diet for at least 18 months. *In model P[a] Z-score was unadjusted.
